# Position paper on current aspects of sponsoring in accredited CME

**DOI:** 10.1080/21614083.2017.1312062

**Published:** 2017-04-28

**Authors:** Daiana Stolz, Reinhard Griebenow, Andre Tichelli, Andrew J. Ullmann, Richard W. Costello, Lampros Michalis, Margarita Guenova, Sandy Sutter, Fabiola De Andrade, Robert Schaefer

**Affiliations:** ^a^ on behalf of the European Board for Accreditation in Pneumology (EBAP), Lausanne, Switzerland; ^b^ Department for Pneumology, University Hospital Basel, Basel, Switzerland; ^c^ on behalf of the European Board for Accreditation in Cardiology (EBAC), Cologne, Germany; ^d^ Municipal Hospital Cologne-Merheim, Department II for Internal Medicine II, University of Cologne, Cologne, Germany; ^e^ on behalf of the European Board for Accreditation in Haematology (EBAH), The Hague, Netherlands; ^f^ Department for Haematology, University Hospital Basel, Basel, Switzerland; ^g^ on behalf of the European Board for Accreditation in Infectious Diseases (EBAID); ^h^ Department II for Internal Medicine, University Hospital Wuerzburg, Wuerzburg, Germany; ^i^ Royal College of Surgeons in Ireland, Education and Research Centre, Beaumont Hospital, Dublin, Ireland; ^j^ Department II for Cardiology, University Hospital Ioannina, Ioannina, Greece; ^k^ National Specialised Hospital for Active Treatment of Hematological Diseases, Sofia, Bulgaria; ^l^ EBAP, Lausanne, Switzerland; ^m^ EBAH, The Hague, Netherlands; ^n^ EBAC, Cologne, Germany

**Keywords:** European Specialty Accreditation Boards, independent CME, sponsoring, transparency, professional congress organiser (PCO), medical education company, information material

## Abstract

This position paper is the result of a collaborative approach of several European Specialty Accreditation Boards (ESABs) and, has been stimulated by their current experience in accreditation regarding roles and responsibilities assumed by sponsors of accredited continuing medical education (CME). The suggestions made in this paper aim to preserve the fundamental principle in CME accreditation that the physician in charge of the programme has sole responsibility for the selection of topics, speakers, content and format, as well as mode of presentation, and that sponsors will under no circumstances interfere with this principle. This is considered as a responsibility of an individual physician (or physicians), which cannot be delegated, even in part, to third parties. This responsibility has been extended to include all communication before and after the event. The paper also identifies undecided issues, about which ESABs are committed to elaborate proposals in the future.

For more than 15 years, European Specialty Accreditation Boards (ESABs) have striven to provide an element of quality assurance to the European medical community by offering accreditation of continuing medical education (CME) and continuing professional development (CPD).

Recent developments in the field of organisation and sponsoring of CME have stimulated this position paper, which does not replace any of the rules in force, but seeks to amend and specify existing principles and rules in the accreditation of CME.

For accreditation of CME/CPD programmes, major accreditors, including the ESABs, have mandated that the physician in charge of the programme (“physician course director”, “physician organiser” etc.) has the sole responsibility for selection of topics, speakers, content and format, as well as mode of presentation, and that sponsors will under no circumstances interfere with this principle. We consider this a responsibility of an individual physician (or physicians), which cannot be delegated, even in part, to third parties, including, in particular, professional congress organisers (PCOs), so-called medical education companies (PCOs with a focus on organisation of CME/CPD) or sponsors.

Sponsoring, by definition, needs something in return from the beneficiaries of sponsoring. This is achieved by making sponsoring transparent through mentioning the name and logo of the sponsor

1. on the last page of the programme (live events),
2. on the last slide (e-learning materials, links to the sponsor not allowed), or
3. at the end of the article (CME in print media).

In addition, the sponsors should be offered the opportunity to present their promotional material at a site outside the room in which the CME/CPD activity takes place (and all other rules issued by ESABs still apply).

ESABs also encourage mentioning the amount of money provided by the sponsor together with the name and logo at the sites indicated above. Further acknowledgements (product trade names, etc.) are not allowed, and use of terms like “platinum sponsor”, etc., instead of exact amounts, is discouraged. Sponsors should not claim any further return on sponsoring.

All communication to (potential) participants should be totally unambiguous and should emphasise the complete independence of the accredited activity.

In this regard, ESABs

1. strongly discourage the use of sponsors’ services for registration of participants (website, organisation secretariat),
2. do not allow promotion of an accredited activity on the website of the sponsor(s) (though hyperlinking is allowed), and
3. allow sponsor(s), as well as PCOs, to distribute CME programmes to potential participants, but consider sending programmes by post, showing the logo of the sponsor to be inappropriate.

It is mandatory that all accredited CME/CPD is available to the whole medical community. However, should any accredited live event address a highly selected audience, exclusion of participants must always be based on a physician (organiser)-to-physician (participant) interaction, and sponsors or PCOs must not play any role in this process.

Transparency is a key prerequisite for identification of potential sources of bias based on conflicts of competing interests. Thus, ESABs strongly encourage all initiatives that aim to implement strategies for achieving timely, comprehensive and relevant transparency with regard to all stakeholders involved in design, planning and delivery of CME/CPD.

Currently, recommendations on how to declare and manage conflicts of interest focus on physicians in their role as members of an organising committee/congress programme committee or faculty member. Major accreditors all require that interests of members of this group should be comprehensively declared and, in addition, offer forms to be used for declaration [1]. Furthermore, providing no declaration at all is one of the few reasons for exclusion from a faculty or other position involved in the planning and/or delivery of CME/CPD (besides being an employee of a commercial interest, including PCOs).

In contrast, identification and management of interests of organisations has received much less attention. ESABs would like to stimulate a discussion on how to design declarations of conflicts of interest to be used by all organisations involved in hosting, planning and delivery of CME/CPD, including PCOs, and academic and non-academic medical centres (some of which have institutional cooperation agreements with companies acting as sponsors in CME/CPD). For the time being, ESABs claim the right to see all contracts between PCOs and sponsors or faculty, in order to find out whether agreements have been reached that contravene their accreditation rules. ESABs strongly discourage direct contractual agreements between members of scientific/organising committees and sponsors.

Since ESABs consider mono-sponsored CME/CPD activities to have the highest risk of undue influence by the sponsor, they would like to open a debate on which additional information, obtained from the provider, might demonstrate the independence of the CME programme.

Undecided issues raised in this paper will be further discussed by ESABs to formulate proposals, which might then be implemented in their accreditation procedures. For that purpose, this position paper will be revised on a regular basis.

## Supplementary Material

Declaration_of_interests.pdf

## Notes

This paper has also been endorsed by the Accreditation Council of Oncology in Europe (ACOE).
