# Supplementary material for: Position paper on current aspects of sponsoring in accredited CME
Source: J Eur CME. 2017 Apr 28;6(1):1312062. doi: 10.1080/21614083.2017.1312062 (PMC5843059; doi:10.1080/21614083.2017.1312062)
Supplement: Declaration_of_interests.pdf [file ZJEC_A_1312062_SM2625.pdf]

Declaration of interests:

1. **Prof. Reinhard Griebenow, MD, PhD:**

   Financial: 0

   Nonfinancial: Assistant Medical Director and Head of Training, Department of Cardiology, Angiology and Diabetology, Municipal Hospital Cologne (Merheim), University of Cologne, Germany

   President, UEMS Cardiology Section

   Chairman of the Board, ECSF

   Chairman, EBAC Advisory Committee

   Head, Academy for Training and Education, Chamber of Physicians North-Rhine, Duesseldorf

   Member of the Board, Chamber of Physicians North-Rhine

   Member, Standing Committee for CME, German Medical Association

   Member, Education Committee, German Cardiac Society

   Member, Editorial Board, JECME

2. **Prof. Lampros Michalis, MD, PhD**

   Financial: 2

   Nonfinancial:

   - **Member of the British Medical Association, UK**

- **Member of the Royal College of Physicians, UK**

- **Member of the British Society of Echocardiography**

- **Regional Representative of the British Society of Echocardiography for the West Midlands region**

- **Member of the Greek Cardiac Society**

- **Member of the British Cardiac Society**

- **Member of the Working Group of the Greek Cardiac Society for Interventional Cardiology**

- **Fellow of the European Society of Cardiology (FESC)**

- **Member of the Scientific Councils of Atherosclerosis/ Thrombosis and Vascular Biology of the American Heart Association (AHA)**

- **Member of the International Society of Endovascular Specialists**

- **National Representative of the Hellenic Cardiology Society to the UEMS Cardiology Section.**

- **Professional Member of the American Heart Association and American Stroke Association.**

- **Member of the Greek Society of Cardiovascular Research**

- **Member of the Working Group of Aortic Disease and Peripherals of the Hellenic Cardiology Society**

- **Director of the Executive Committee of the Michaelideion Cardiac Center**

- Member of the Board of Directors of the Hellenic Cardiological Society

- Appointed Member of the Hellenic Cardiological Society

- Nucleus Member of the Working Group of Invasive Cardiology of the Hellenic Cardiology Society

- President of the working group of Invasive Cardiology of the Hellenic Cardiology Society

- Vice president of the Scientific Committee of the Greek College of Cardiology

- Member of the European Board for Accreditation in Cardiology Reviewing Committee

- Member of the Executive Board of the University Hospital of Ioannina

- Secretary of the UEMS cardiology section – Vice President of EBAC

- Member of the Board of the ECSF (European Cardiology Section Foundation)

- Chief Editor of the Continuing Education of Cardiology Journal of the Greek College of Cardiology

- Member of the Public Relations Committee of the European Society of Cardiology

- Substitute member of the Committee responsible for the examinations certifying the Completion of Specialist Training in Cardiology in the region of Epirus

- In charge of the Scientific Committee of the Research Laboratory of the University Hospital of Ioannina

- Member of the Committee responsible for the examinations certifying the Completion of Specialist Training in Cardiology in the region of Epirus

- Substitute member of the Educational Committee of the Greek National Board of Health

- Member of the Greek NHS Committee for the Evaluation of Cardiac Centers capability in regards with the implantation of pacemakers and defibrillators.

- Member of EBAC Advisory Committee

- Member of the International Scientific Committee organizing actions for the attraction of Scientific Meetings in Greece

- Director of 2nd Cardiology Department University Hospital of Ioannina

- Committee of education of the Greek NHS (substitute member)

- Vice President of the Medical School of Ioannina

3. Robert Schäfer, MD

Financial: 0

Nonfinancial: CEO, EBAC

President, General Assembly German National Ass. for Occupational Health and Safety in Medicine and Welfare,

Member, Board of Children Safety Foundation NRW, Germany

Member, Board of Trustees KKLE Hospitals, Kleve, Germany

CEO Chamber of Physicians North-Rhine, Duesseldorf, Germany, retired

4. Prof. Daiana Stolz, MD, PhD

Financial: 3

Grants/research support: Thermo-Fisher Scientifics, Swiss National Foundation, Internal Medicine Research Grant, University Hospital Basel, ResMed, Weinmann AG, Pan Gas AG, Novartis, Boston Scientifics, Curetis

Speakers honoraria or consultations fees: Boehringer Ingelheim, Almirall, Novartis, Glaxo, Astra, Bayer

Non-financial:

Clinic for Pulmonary Medicine and Respiratory Cell Research, University Hospital Basel, Basel, Switzerland

Member of the steering board of the Swiss Respiratory Society, since 2010

Past president of the European Board of Accreditation in Pneumology (2014-2016)

Member of the Editorial Board CHEST, since 2010

Member of the Pneumology Board Committee, Swiss Respiratory Society, since 2013

Past Co-chair of the Training Centre Accreditation Committee, HERMES 2014-2016

Member of the Educational Committee, American Thoracic Society, 2015-2016

Member of Long Range Planning Committe, Assembly 10, European Respiratory Society, since 2015

Fellow of the College of Chest Physician, since 2015

Member of the European Respiratory Society Fellowships &

Awards Working Group, since 2015

Chair, International Affairs, Swiss Respiratory Society, since 2015

Member of the Lung function Committee, Swiss Respiratory Society, since 2015

Chair, Organizing committe, CHEST/SGP 2017, Basel, Switzerland

President Post Graduate Training Committee, Swiss Pneumology Society, since 2016

Education Council elect, European Respiratory Society, since 2016

5. Richard Costello, MD:

Financial: 0

Non-financial:

President, European Board for Accreditation in Pneumology (EBAP)

Member, Education Council, European Respiratory Society

Director of Research Royal College of Physicians in Ireland

6. Sandy Sutter:

Financial: 0

Non-financial:

Employee, European Board for Accreditation in Pneumology (EBAP)

Employee, European Respiratory Society

7. **Prof. André Tichelli, MD**

   Financial: 0

   Non-financial:

   Chairman of the Board, EBAH

   President of the commission of postgraduate and continuing education of the Swiss Society of Hematology (SSH)

   Member of the committee of the Swiss Society of Hematology (SSH)

   Member, Editorial Board, Biology of Blood and Marrow Transplantation

   Member of the Educational Committee of the European Hematology Association (EHA)

   Past chair of the Late Effects Working Party of the European Blood and Marrow Transplantation (EBMT)

   Past president of the Swiss Society of Hematology (SSH)

8. **Prof. Margarita Guenova, MD, PhD**

   Financial: 3

   Grants/research support: Novartis, Sanofi, Bulgarian National Research Fund

   Speakers honoraria or consultations fees: Novartis, Amgen,

Sanofi, Roche, Abvie, Gilead

Non-financial:

Laboratory of Haematopathology and Immunology, National University Specialised Hospital for Active Treatment of Hematological Diseases, Sofia, Bulgaria

Visiting Professor in Haematology, Medical University - Sofia

Member of the Board of the Bulgarian Medical Society of Hematology, since 2007

President of the Bulgarian Medical Society of Hematology, since 2011

Member of the Editorial Board "Hematology" (Journal of the Bulgarian Medical Society of Hematology), since 2011

Member of the Executive Committee of the European Association of Hematopathology, since 2016

Member of the Curriculum Committee of EHA (European Hematology Association)

Member of the Good governance Committee of EHA (European Hematology Association)

Chair-elect of EBAH (European Board of Accreditation on Hematology)

9. Fabíola de Andrade:

Financial: 0

Non-financial:

Employee, European Hematology Association (EHA)
